# Engaging Physicians and Systems to Improve Hepatitis C Virus Testing in Baby Boomers

**DOI:** 10.3390/healthcare11020209

**Published:** 2023-01-10

**Authors:** Susan T. Vadaparampil, Lindsay N. Fuzzell, Julie Rathwell, Richard R. Reich, Richard Roetzheim, Anna R. Giuliano

**Affiliations:** 1 Department of Health Outcomes and Behavior, H. Lee Moffitt Cancer Center, Tampa, FL 33612, USA; 2 Department of Cancer Epidemiology, H. Lee Moffitt Cancer Center, Tampa, FL 33612, USA; 3 Center for Immunization and Infection Research in Cancer, H. Lee Moffitt Cancer Center, Tampa, FL 33612, USA; 4 Biostatistics and Bioinformatics Shared Resource, H. Lee Moffitt Cancer Center, Tampa, FL 33612, USA; 5 Department of Family Medicine, University of South Florida, Tampa, FL 33620, USA

**Keywords:** hepatitis C virus, electronic health records, clinical decision support system, prevention

## Abstract

**Simple Summary:**

Millions of people in the United States are chronically infected with the hepatitis C virus (HCV). Baby boomers have substantially higher HCV infection rates than other age groups. Clinical practice guidelines recommend HCV testing in baby boomers, but testing rates are low. We developed and tested an intervention to increase orders for HCV testing that included electronic health record supports and a physician education session to improve HCV physician knowledge in one Florida academic health system. During the intervention, for providers that viewed a pop-up alert in the electronic health record, HCV test ordering increased. The brief physician education intervention improved HCV knowledge and increased self-efficacy in the knowledge of HCV risk factors. These findings suggest electronic health record and physician education interventions hold promise for increasing HCV testing rates.

**Abstract:**

Approximately three million people in the United States have been exposed to the hepatitis C virus (HCV), with two-thirds of these having chronic HCV infection. Baby boomers (those born 1945–1965) have nearly five times the prevalence of HCV infection compared with other age groups. Despite clinical practice guidelines that recommend HCV testing in baby boomers, the testing rates remain low. We developed and tested a multilevel intervention to increase orders for HCV testing that included integrated clinical decision support within the electronic health record (EHR) and a physician education session to improve HCV physician knowledge in one Florida academic health system. In the year prior to the intervention, test order rates for encounters with baby boomers was 11.9%. During the intervention period (August 2019–July 2020) for providers that viewed a best practice alert (BPA), the ordering increased to 59.2% in Family Medicine and 64.6% in Internal Medicine. The brief physician education intervention improved total HCV knowledge and increased self-efficacy in knowledge of HCV risk factors. These findings suggest that interventions at the system and physician levels hold promise for increasing HCV testing rates. Future studies are needed to evaluate this intervention in additional clinical settings and to test the benefit of adding additional intervention components that are directed at patients.

## 1. Introduction

Approximately three million people in the United States have been exposed to the hepatitis C virus (HCV), [1] with 65–75% becoming chronically infected [2]. Half of those with HCV are unaware of their status and therefore do not obtain treatment to achieve a sustained curative virologic response [3]. Between 2012 and 2013, the U.S. Preventive Services Task Force (USPSTF) and the Centers for Disease Control and Prevention (CDC) recommended all baby boomers (born between 1945 and 1965) be screened for HCV infection based on higher prevalence of the infection compared with other age groups [4,5,6]. Recently, both the USPSTF and CDC issued universal HCV testing recommendations for all adults [7,8]. Healthcare provider recommendation for HCV testing is a critical first step toward ensuring orders and completion. However, HCV testing nationally and in Florida is underutilized. A recent study of seven health systems in Florida found that in 2016–2017, providers ordered HCV tests for only 6.7% of their baby boomer patients, and 89.7% of those patients completed HCV testing, resulting in a functional HCV test completion prevalence of only 6% [9].

Electronic Health Record (EHR)-based Clinical Decision Support System (CDSS) interventions may improve HCV test ordering [10,11,12,13]. The CDSS provide clinicians and staff with knowledge and person-specific information, intelligently filtered and presented at appropriate times, to enhance healthcare delivery and outcomes [14]. Multiple studies have documented the benefits of CDSS integrated into EHRs for a variety of clinical outcomes [15]. Interventions utilizing these practice alerts consistently demonstrate double digit increases in HCV testing [10,11,12,13]. Automated order sets for recommended follow-up care also improve linkage to care after positive HCV test results [16,17]. EHR-based interventions that incorporate provider education may further improve HCV testing [18,19,20]. One quality improvement initiative (QI) utilizing provider education increased HCV testing rates for baby boomers by 34% (25.9% to 59.9%) over a six-month period [21].

Given the evidence described above supporting these approaches, we implemented an EHR-based QI intervention with an in-person physician education session, combined with an integrated CDSS within the EHR of one academic health system in West Central Florida to increase orders for HCV testing in baby boomers. We report the HCV test order rates, test completion rates, and orders per encounter for baby boomers during the intervention period compared to a comparable time period prior to the intervention. We also describe follow-up tests ordered following the SmartSet suggestions for patients with current HCV infections. Finally, we detail pre-post data related to the provider educational intervention.

## 2. Method

We conducted and evaluated a QI project with eligible physicians in three primary care departments of an academic health system: Family Medicine (FM), Internal Medicine (IM), and Internal Medicine/Pediatrics ([IMP] which sees both adult and pediatric patients). The project consisted of physician education, and an EHR-based Best Practice Alert (BPA) suggesting HCV antibody screening with reflex to RNA and genotype testing for the baby boomer patients, with a suggested SmartSet for those who test positive. To assess changes in HCV testing, we compared the EHR data for patient visits from August 2019 to July 2020 (intervention period) with comparable patient visits from August 2018 to July 2019 (pre-intervention/comparison period). The primary outcome was the percentage of HCV tests ordered per visit during each time frame. This study was approved by institutional IRB (Pro00039312; Pro00039969).

### 2.1. Academic Health System and Data Inclusion

We utilized a single academic health system—one of the largest physician groups in West Central Florida with over 900 healthcare professionals and 555,000 office visits in fiscal year 2019. This health system has used Epic as the EHR since August 2015. Clinical data from 1,130,527 unique patients (based on billed encounters) are stored in a searchable data warehouse that can be downloaded for health services research. We included encounters with primary care providers in the three included departments (FM, IM, IMP).

### 2.2. Intervention Development

The intervention was developed leveraging Cabana’s Practice Improvement Framework [22]. Specifically, the physician education component targets knowledge as a barrier, while the CDSS addresses health system factors that may limit physician screening orders and follow-up care [22].

### 2.3. Systems Intervention

The standard of care in the health system consisted of a health maintenance tab located within the patient’s chart listing all screenings (including HCV), lab work, etc., due for each patient based on age, gender, and/or health condition. The physician or any member of the healthcare team (e.g., nurse, advanced practice professional, medical assistant) could view all the recommendations by clicking on the tab. We partnered with the healthcare system’s Clinical Decision Support Committee to develop the systems intervention, following their established processes for developing, testing, and refining CDSS. The systems intervention included the CDSS tools available in Epic (1) a Best Practice Alert (BPA) and (2) a SmartSet. The BPAs are reminder tools (e.g., a pop-up alert) that notify physicians an action is needed based on a pre-specified single, or group of, patient characteristics [23,24,25,26]. For the current QI project, the BPA was triggered only when a physician placed any order (e.g., lipid panel, HbA1C; mammogram, urinalysis, etc.) in the EHR for a patient born between 1945 and 1965. The pop-up noted the reason for the suggested HCV testing (i.e., high-risk age group) and asked the physician to accept the order for an HCV antibody screen with reflex to RNA and genotype testing. If they chose not to place the order, they were asked to acknowledge a reason, which could include “incorrect alert”, “patient declined”, “already completed”, “not addressed today”, or they could close the window without selecting a reason. A SmartSet is a pre-configured group of automated orders for specific diagnoses or patient groups, which can be placed with a single click, and are effective at increasing HCV testing rates [27]. For those who tested positive, the automated order set offered the opportunity to place orders for: Fibroscan to assess cirrhosis, lab work for co-infections (hepatitis A virus (HAV), hepatitis B virus (HBV), human immunodeficiency virus (HIV)), comprehensive metabolic panel (CMP), comprehensive blood count (CBC), opiate drug screen, and referral to a gastroenterology specialist. The providers could select as many, or as few, of the orders as they considered appropriate.

### 2.4. Physician Education Intervention

The physician intervention component consisted of a one-hour in-person interactive educational and skill building session for physicians in the Department of Family Medicine (while physicians in the two other participating primary care departments of Internal Medicine and Internal Medicine/Pediatrics did not receive the physician education intervention). The session was led by a physician educator and addressed HCV knowledge gaps including modes of transmission, consequences of infection, and the need for HCV testing among high-risk populations, baby boomer specific testing recommendations, and an overview of direct acting antivirals. Physicians were shown aggregate department-specific HCV testing rates. Finally, the session introduced the CDSS tools. Prior to and immediately following the educational session, physicians completed knowledge and self-efficacy assessments. Four multiple choice items assessed physicians’ knowledge, scored as correct or incorrect, while two self-efficacy items asked physicians to rate their confidence in HCV risk factor knowledge and ability to recommend and place HCV testing orders. Four items captured impressions of the training. See Table 1. Because this project was being conducted as part of a quality improvement effort, the IRB determined that physicians informed consent could be waived.

### 2.5. Variable Description and Statistical Analysis

We assessed patient demographic characteristics, including age, race/ethnicity, and preferred language. We identified the HCV tests ordered from all eligible encounters from August 2019 to July 2020 (intervention period) to comparable patient visits from August 2018 to July 2019 (comparison period), and the HCV tests completed of those ordered. We assessed percentages and 95% confidence intervals of the HCV tests ordered and completed during the comparison period for all three participating departments. We then estimated the HCV test order and completion rates during the intervention period, comparing FM (received the physician education intervention) to IM and IMP combined (did not receive the physician intervention). These rates were compared using chi-square tests. Additionally, we display the percentages of HCV orders per encounter in which standard of care or BPA was viewed as a function of time to visualize impacts of the COVID-19 pandemic, which started to impact clinic visits in March 2020 on numbers of encounters. Furthermore, for HCV RNA-positive patients, we assessed the provider selection of orders that were suggested by the SmartSet and report date(s) of test orders. Finally, we conducted paired samples *t*-tests to assess the change in physician knowledge and self-efficacy from pre to post educational intervention and describe the overall physician impressions of the training. Statistical analyses were conducted in SAS 9.4.

## 3. Results

**Figure 1:**
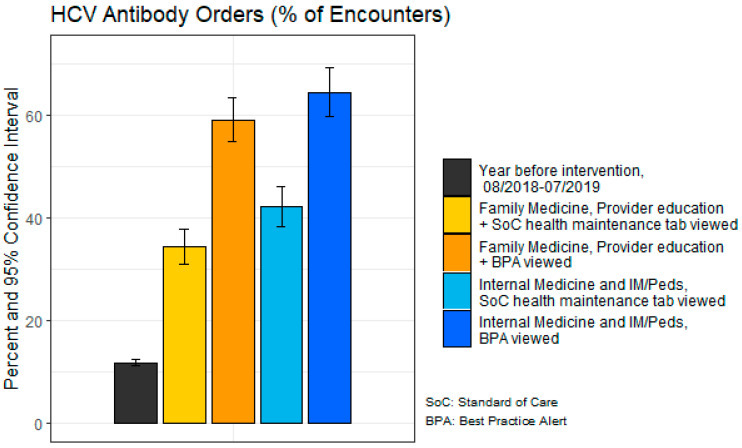
Hepatitis C virus (HCV) antibody orders and Clinical Decision Support System (CDSS) component implemented for Family Medicine (FM) and Internal Medicine (IM), and Internal Medicine/Pediatrics (IMP) departments.

Table 2 displays the age, race/ethnicity, and preferred language of the 1753 patients who participated in eligible encounters during the intervention period, as well as for 6371 baby boomers seen during similar encounters in the comparison period. Encounters from 79 providers were included in the intervention period. Patients in the intervention period were ~64 years old (M = 63.8, SD = 5.8), majority white (67.1%), and preferred English (97.2%). Patients in the comparison period were similar in age (M = 63.1, SD = 5.87), race (68.4% white), and language preference (96.6% English). Encounters from 90 providers were included in the comparison period. Figure 1 displays the HCV test orders and completions by physician educational intervention participation, CDSS type, and department. In the year prior to the intervention (comparison period), only 11.9% of encounters with baby boomers included an order for an HCV test, with 66.5% of those ordered tests completed by patients, resulting in 7.9% actually tested. Of the 2263 eligible encounters during the intervention period, 1070 HCV tests were ordered (47.3%) and 581 HCV tests were completed (25.7%). The encounters during the intervention period most frequently included those in which both the standard of care health maintenance tab and BPA were viewed (50.09%, *n* = 2263); during 38.45% of encounters only the standard of care health maintenance tab was viewed, contrasted with 11.46% in which only the BPA was viewed. During the intervention period, in encounters in which only the standard of care health maintenance tab was viewed, 34.4% and 42.3% of encounters in FM and in IM/IMP combined, respectively, resulted in orders placed. More orders were placed in encounters in which the BPA fired was higher, ranging from 59.2% for FM to 64.6% for IM and IMP combined (Figure 1). The difference between the overall HCV test orders in the eligible encounters during the comparison period contrasted with those in which the BPA was viewed during the intervention period was statistically significant (*p* < 0.0001).

**Figure 2:**
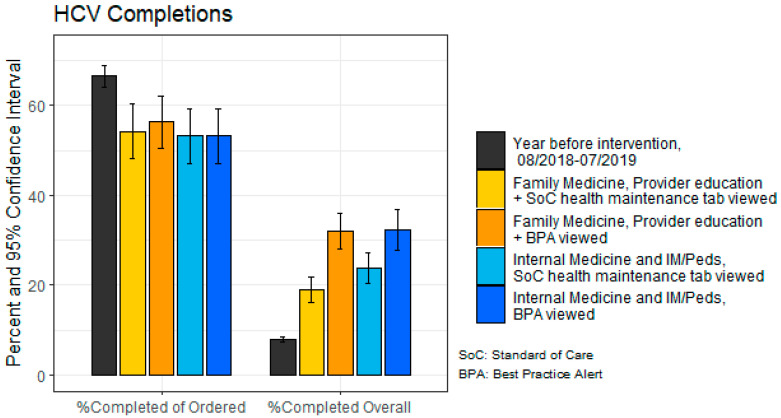
Hepatitis C virus (HCV) antibody test completions of ordered and completions overall and Clinical Decision Support System (CDSS) component implemented for Family Medicine (FM) and Internal Medicine (IM), and Internal Medicine/Pediatrics (IMP) departments.

Within a department, ordering rates were higher among providers who did, compared with those who did not, view the BPA (59.2% vs. 34.4% within Family Medicine; 64.6% vs. 42.3% within Internal Medicine and Internal Medicine/Pediatrics) (Figure 1). During the intervention period, test *completion* rates were similar across physician education intervention participation, CDSS type, and department, with rates between 53.3% to 56.3% (Figure 2). Overall, the percentage of ordered tests that were completed was significantly higher in eligible encounters the year *prior* to the intervention (comparison period) compared with those in which the BPA was viewed during the intervention period (*p* < 0.0001). Although the completion percentage was lower during the intervention period (54.3% completed of 47.2% ordered in the intervention period versus 66.5% completed of 11.9% ordered in the comparison period), the percentage of the entire sample actually tested was higher due to the higher order rates (581 completed of 1753 patients (33%) during the intervention period versus 1068 completed of 6371 patients (17%) during the comparison period). When examining by visits during the intervention period, the rate of actual screening was 25.7% of visits (32.2% when the BPA fired; 21.1% when it did not).

**Figure 3:**
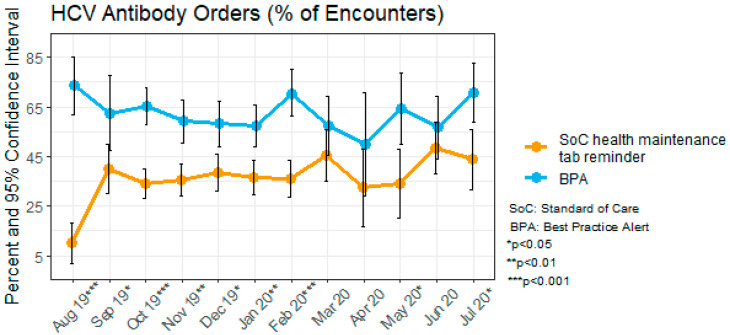
Hepatitis C virus (HCV) antibody orders per encounter by Clinical Decision Support System (CDSS) type.

Figure 3 displays rates of HCV test orders as a function of time by CDSS type (BPA versus standard of care health maintenance reminder). Although the numbers of total encounters dropped, beginning in March to April of 2020 due to the COVID-19 pandemic (M = 247 during August 2019 to February 2020; M = 114 during March 2020 to July 2020), encounters where a BPA appeared had a higher rate of test orders in every month than those for which standard of care health maintenance tab was viewed. These higher rates were statistically significant (*p* < 0.05) in each month prior to the pandemic period and in two of four months (May and July) during the pandemic period (beginning March 2020). The statistical power was lower and confidence intervals larger during the pandemic period due to the lower numbers of encounters overall.

Ten patients (10 of 581 = 0.017 or 1.7%) tested RNA-positive for the HCV during the intervention period. Supplementary Table S1 displays follow-up tests ordered of those suggested by the SmartSet for those patients. Tests ordered by a provider are indicated by date(s) of order. Blank cells indicate no order placed. Of the ten HCV RNA-positive patients, there were no opiate screen orders, only one Fibroscan order, and three referral orders to gastroenterology. The most common were orders for CMP and CBC. For coinfection, the most common were HBV orders, followed by HIV, and HAV. Overall, two patients (patients B and E) received only one follow-up order and two received two follow-up orders (C and G); six patients were ordered a larger group of follow-up orders (at least five orders), indicating variation in the provider selection of orders suggested by the SmartSet.

**Figure 4:**
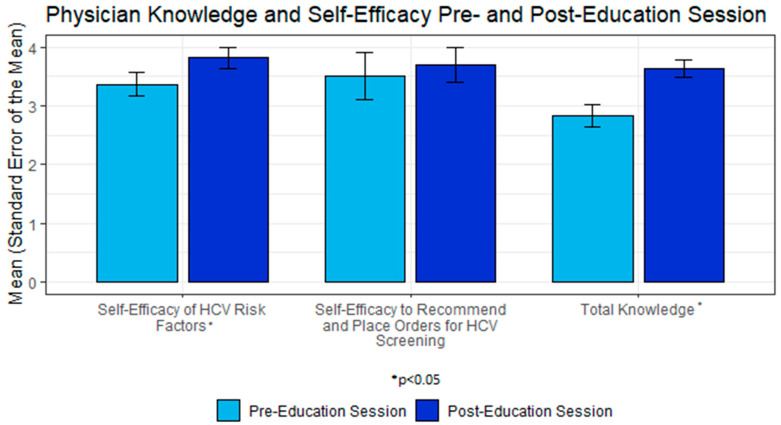
Total hepatitis C virus (HCV) knowledge, self-efficacy of HCV risk factor knowledge, and self-efficacy to recommend and place orders for HCV screening prior to the physician education session and following the physician education session (*n* = 11).

In total, 11 (of 15 possible) physicians in the Department of Family Medicine attended the HCV education session; the mean total knowledge increased from pre- to post-education session (Pre: M = 2.82, SD = 0.60; Post: M = 3.64, SD = 0.50; Cohen’s d = 1.2; Figure 4). Overall, there was a statistically significant improvement in total HCV knowledge from pre-test to post-test (t(10) = −3.11, *p* = 0.011). Self-efficacy in knowledge of HCV risk factors significantly increased from pre-test to post-test (t(10) = −2.89, *p* = 0.016). Despite the small mean increase (3.50 to 3.70), there was no statistically significant increase in self-efficacy to recommend and place orders for HCV testing. All physicians who participated in the training rated the information provided about the risk factors for HCV infection, HCV screening and post-diagnosis HCV evaluation and treatment, HCV BPA for screening, and HCV infection order sets as either extremely or very useful. A total of 10 of 11 participants felt that the information presented about the Health Maintenance Tab was either extremely or very useful.

## 4. Discussion

We developed a physician- and systems-level intervention to increase orders for HCV testing in baby boomers in one health system in Florida. The HCV test order rates increased significantly compared with the year prior to the intervention, and physician HCV knowledge and in self-efficacy in the knowledge of HCV risk factors improved.

In the year prior to the intervention, ~12% of encounters with baby boomers included an order for an HCV test, but during the intervention period, test order rates for encounters with baby boomers in which a BPA appeared were ~59% in Family Medicine and ~65% in Internal Medicine and Internal Medicine/Pediatrics, and accordingly were ~34% (FM) and ~42% (IM/IMP) for those who viewed the standard of care. This indicates the BPA was an effective prompt that reminded physicians to place HCV test orders.

Both viewing the standard of care health maintenance tab and the BPA alert resulted in higher ordering in IM physicians compared to FM physicians. In the years prior to the intervention period, FM clinics overall more frequently ordered HCV testing, but this could have been due to efforts of a physician champion in the department (mean ~25% HCV orders in FM, ~15% IM, ~3% IM/IMP). Therefore, any increases in ordering may have resulted in only a modest increase in the overall percentage of tests ordered, since ordering was already high at the outset.

Notably, the percentages of HCV tests completed were actually lower across the department and CDSS type when compared with the year prior to the intervention. This is likely a result of the COVID-19 pandemic, which started midway through our intervention period. Data from a large clinical laboratory indicated that HCV antibody test volume drastically dropped by 59% by April 2020, with a near rebound to pre-pandemic levels by July 2020 [28]. The low HCV test completion rate may also indicate that while targeting physician behavior is important, another potential intervention target may be at the patient level.

For orders placed from those suggested by the SmartSet, there was a range of variability in the tests ordered for HCV-positive patients. Although there were only ten HCV-positive patients, our results give early insights into how physicians utilize the suggested SmartSets. The physicians appeared to use their clinical judgment to select appropriate follow-up tests. For instance, there were no orders for opiate drug screens, an example of the providers assessing the individual patient’s age/history and determining no current need for a drug screen. The recent spikes in opioid-related HCV infections primarily occurred among young adults, rather than baby boomers [29]. For at least two patients, we have limited understanding of the co-infection testing and referrals (likely due to the timing of when they received a positive test). For six patients, the physicians placed the majority of the SmartSet follow-up orders. Overall, Fibroscan and gastroenterology referrals were infrequent, although they could have occurred outside of the study period or at another health system; few gastroenterology referrals could be indicative of desire to manage HCV cases in primary care. Ideally, a provider would select all of the orders suggested by the SmartSet, as they were designed to mirror the clinical practice guidelines for follow-up after a positive HCV RNA test. However, given that providers have varying levels of comfort in managing HCV positive patients, we would expect providers to select all of the imaging and labs in the SmartSet, or refer to a gastroenterology colleague.

Our brief physician education session showed improved total HCV knowledge and increased self-efficacy in the knowledge of HCV risk factors. Notably, the physicians who participated in the education session were from FM, the department which consistently had lower rates of ordering compared to IM departments during the intervention period. This may indicate that our intervention session included helpful information about general HCV topics but should be expanded to adequately cover EHR ordering and CDSS reminders and automated order sets. Taken together, these findings indicate that the system and physician education intervention provided a benefit to HCV testing, but that a patient intervention component may be helpful in improving test orders. Future research should evaluate the role of a patient component that addresses improving HCV knowledge, as well as engagement with their care surrounding testing for HCV infection. Patient knowledge could be improved through printed or interactive educational materials, and interventions could address patient engagement and activation through tools, such as Question Prompt Lists, a useful aid for increasing patient engagement and health outcomes in other contexts [30].

This work has several unique strengths. We developed and implemented an EHR intervention with CDSS tools in a large health system, including pop-up reminder alerts for HCV testing and order sets for those that tested positive. The use of the EHR to extract HCV test order and completion rates is an important strength and eliminates the need to rely on self-reporting of test ordering and/or completion. The inclusion of tests ordered of those suggested by the SmartSet is a unique component of this manuscript, highlighting the need for further investigation into how providers make decisions around follow-up orders for HCV-positive patients, and for capturing the cascade of care and completion of treatment for those positive at initial screening. Finally, we piloted a physician HCV education session with a small group of primary care providers, a unique addition to the systems intervention.

However, findings should be tempered by a few limitations. First, we were only able to pilot the physician education component in one department (Family Medicine) in a single health system, which limits the broad applicability and effectiveness in improving knowledge and self-efficacy, and our ability to determine the synergistic effects of the physician education and EHR intervention, across all primary care departments. Next, the requirement of the pop-up to indicate why the provider chose not to place an order, with opportunity to indicate reasoning, may have had an impact on provider behavior. We are unable to ascertain the impact of this prompt for reasoning on the test order rates. In addition, the standard of care reminders only appeared during encounters in which physicians clicked on the health maintenance tab, and BPA pop-ups only appeared during encounters in which physicians placed an order. If a patient attended an encounter in which they did not need labs or other tests ordered at that visit, the BPA pop-up did not appear, effectively eliminating these opportunities for an HCV test ordering during an encounter. Similarly, there were encounters in which physicians neither placed an order nor checked the health maintenance tab; we were unable to include these types of encounters in our analyses. In addition, during the implementation of the systems intervention, we discovered nurses, medical assistants, and other medical team staff often place orders on behalf of physicians for laboratory and other types of testing prior to or during encounters, but BPAs were not programmed to fire for these other members of the healthcare team as part of our intervention. This may also have resulted in missed opportunities for HCV test orders and should be considered in the development of future interventions. Finally, we present limited data on follow-up tests ordered, as suggested by the SmartSet for ten patients who had active HCV infections. Although we include the test order date for the relevant follow-up orders, without manual medical record review [31], we are unable to determine if these orders led to the treatment and clearance of HCV. Additionally, the study period was limited to one year and data extracted about follow-up orders may not be complete as orders could have occurred outside of the study window.

## 5. Conclusions

Our findings reinforce the importance of EHR interventions for improving HCV testing and highlight the potential for physician education sessions to reinforce knowledge about HCV risk factors, testing guidelines, and treatment. Future work developing patient-facing interventions to complement systems- and physician-level components may be especially useful, and work teasing out the individual and combined effects of intervention components may guide researchers to determine how these pieces may be tailored for specific health systems and practices. This work has the potential to increase the identification of HCV and reduce the occurrence of hepatocellular carcinoma across adult populations.

## Figures and Tables

**Table 1:** Physician knowledge, self-efficacy, and training impression assessment items and response options.

Domain	Item	Scale or Response Options
Self-efficacy	How confident are you in your knowledge of HCV risk factors?	5-point scale from extremely confident to not at all confident
	How confident are you in your ability to recommend and place orders for HCV screening?	5-point scale from extremely confident to not at all confident
Knowledge	HCV is the leading cause of which type of cancer?	Bladder cancer **Liver cancer** Cervical cancer Lung cancer
	Which age cohort is at highest risk of HCV infection?	Those born after 1985 Those born between 1966 and 1985 **Those born between 1945 and 1965** Those born before 1945
	After HCV diagnosis, co-testing should be conducted for…	HIV **HIV, Hepatitis A, and Hepatitis B** HIV, Hepatitis A, and HPV HIV and HPV
	Antiviral treatment for HCV (without cirrhosis) typically takes about…	4 weeks 6 weeks 8 weeks **12 weeks**
Impressions of training	How useful was the information presented about risk factors for HCV infection?	5-point scale from extremely useful to not at all useful
	How useful was the information presented about HCV screening and post-diagnosis HCV evaluation and treatment?	5-point scale from extremely useful to not at all useful
	How useful was the information presented about the HCV Best Practice Advisory (BPA) for screening and the HCV infection order set?	5-point scale from extremely useful to not at all useful
	How useful was the information presented about the Health Maintenance Tab in relation to HCV infection?	5-point scale from extremely useful to not at all useful

Note: Bold text indicates correct response.

**Table 2:** Characteristics of patients in eligible baby boomer encounters during the intervention (*n* = 1753) and comparison periods (*n* = 6371).

Variable	Mean or Frequency	SD or %
Intervention Period		
Age	63.77	5.83
Race/Ethnicity		
Hispanic	123	7.0%
Non-Hispanic Asian	46	2.6%
Non-Hispanic Black	216	12.3%
Non-Hispanic Other	192	11.0%
Non-Hispanic White	1176	67.1%
Preferred Language		
English	1704	97.2%
Spanish	26	1.5%
Other	13	0.7%
Comparison Period		
Age	63.06	5.87
Race/Ethnicity		
Hispanic	465	7.3%
Non-Hispanic Asian	150	2.4%
Non-Hispanic Black	678	10.6%
Non-Hispanic Other	722	11.3%
Non-Hispanic White	4356	68.4%
Preferred Language		
English	6156	96.6%
Spanish	89	1.4%
Other	40	0.6%

## Data Availability

The data generated in this study are not publicly available due to information that could compromise patient privacy but are available upon reasonable request from the corresponding author.

## Supplementary Materials

The following are available online at https://www.mdpi.com/article/10.3390/healthcare11020209/s1, Table S1: Selection and date of follow-up orders as suggested by the SmartSet for patients testing positive for Hepatitis C virus (HCV) during the study period (*n* = 10).
