# Supplementary material for: Engaging Physicians and Systems to Improve Hepatitis C Virus Testing in Baby Boomers
Source: Healthcare (Basel). 2023 Jan 10;11(2):209. doi: 10.3390/healthcare11020209 (PMC9858629; doi:10.3390/healthcare11020209)
Supplement: Supplementary file 1 [file healthcare-11-00209-s001.zip › healthcare-1959932-supplementary.pdf]

Supplementary Table.

Table S1. Selection and date of follow-up orders as suggested by the SmartSet for patients testing positive for Hepatitis C virus (HCV) during the study period (n=10).

**Patient (date of first HCV RNA test)**	**A (9/30/19)**	**B (12/17/19)**	**C (8/16/19)**	**D (8/5/19)**	**E (11/12/19)**	**F (11/8/19)**	**G (10/4/19)**	**H (10/2/19)**	**I (12/17/19)**	**J (10/31/19)**
**Orders from SmartSet**	**Date(s) of orders from SmartSet**									
Fibroscan - Liver elastography	1/30/20									
Ambulatory referral to Gastroenterology				8/5/19			7/16/20	10/11/19		
Hepatitis B virus (HBV) surface antibody, quan, serum plasma	1/30/20			8/5/19		11/8/19		1/30/20		10/31/19
Hepatitis B virus (HBV) core antibody, total, serum/plasma	1/30/20			8/5/19		12/12/19		1/30/20	12/17/19	10/31/19
CBC and automated differential rflx manual diff	9/30/19; 1/30/20	1/20/20	8/16/19; 4/28/20	8/5/19				1/30/20	12/17/19	10/31/19
Comprehensive metabolic panel	9/30/19;		8/16/19;	8/5/19	11/12/19	11/8/19	7/16/20	1/30/20	12/17/19	10/31/19

(CMP), serum	1/30/20		4/28/20							
Hepatitis B virus (HBV) surface antigen, serum/plasm	1/30/20			8/5/19		12/12/19		1/30/20	12/17/19	10/31/19
Hepatitis A virus (HAV) antibody, total, serum/plasma						12/12/19			12/17/19	10/31/19
Human immunodeficiency virus (HIV) 1/2 Antigen/AB, 4th gen, w/ rflx	1/30/20			8/5/19				1/30/20	12/17/19; 2/5/20	10/31/19
Opiate screen, Urine										
